# Complete pathological response of high grade appendicular neoplasm induced Pseudomyxoma Peritonei (PMP) after neoadjuvant intra-peritoneal chemotherapy: A case report

**DOI:** 10.1016/j.ijscr.2020.05.072

**Published:** 2020-06-06

**Authors:** Naveen Padmanabhan, Haruaki Ishibashi, Kazurou Nishihara, Shouzou Sako, Kanji Katayama, Satoshi Wakama, Yasuyuki Kamada, Yutaka Yonemura

**Affiliations:** a Department of Surgical Oncology, Apollo Cancer Insitutes, Chennai, India; b NPO to Support Peritoneal Surface Malignancy Treatment, Japanese/Asian School of Peritoneal Surface Oncology, Kyoto, Japan; c Department of Regional Cancer Therapies, Peritoneal Surface Malignancy Center, Kishiwada Tokushukai Hospital, Kishiwada, Japan; d Department of Surgery, Graduate School of Medicine, Kyoto University, Kyoto, Japan

**Keywords:** Appendiceal neoplasms, Pseudomyxoma peritonei, Cytoreductive surgery, HIPEC, Intraperitoneal chemotherapy, Case report

## Abstract

**Background:**

Pseudomyxoma Peritonei (PMP) is clinical syndrome characterized by mucinous ascites and gelatinous tumor deposits in the peritoneal cavity. Complete Cytoreduction and Hyperthermic intraperitoneal perfusion is the contemporary standard of care for PMP. A novel treatment approach with Intraperitoneal (IP) chemotherapy has been developed for patients with disease not amenable for complete cytoreduction.

**Case presentation:**

A 72 year old lady had PMP arising from high grade appendicular neoplasm with extensive intraabdominal spread not suitable for complete cytoreduction (PCI -19; multiple mesenteric deposits). Novel approach with tumor debulking and Neoadjuvant Intraperitoneal chemotherapy was done. Excellent clinical response was obtained after 12 sessions of IP chemotherapy with cisplatin and docetaxel.

Subsequently she underwent Complete cytoreductive surgery with peritonectomy and Hyperthermic intraperitoneal chemotherapy. Pathological examination of surgical specimens revealed only acellular mucin with no viable tumor cells indicating a complete response.

**Discussion:**

Complete pathological response after IP chemotherapy in extensive PMP is rare. Nevertheless the results are encouraging as the systemic therapy hasn't yielded successful outcomes. IP chemotherapy has the advantage of achieving high intraperitoneal concentrations and down staging the tumor spread.

**Conclusion:**

Neoadjuvant Intra-peritoneal chemotherapy is a promising neoadjuvant strategy in patients who are poor candidates for upfront resection due to extent of disease or performance status, perhaps better than systemic therapy.

## Highlights

• Completeness of cytoreduction is the key factor for long term survival in pseudomyxoma peritonei.
• Neoadjuvant chemotherapy for unresectable cases of PMP has been tried but often with discouraging results.
• Intraperitoneal administration of chemotherapy can result in higher drug concentrations in the peritoneal cavity.
• A seventy two year old lady presented with extensive bulky PMP received laparoscopy, Extensive Intraperitoneal lavage and Intraperitoneal chemoport insertion.
• After 12 sessions of NIPT, she had complete resolution of disease and was treated with complete cytoreductive surgery with peritonectomies and Hyperthermic Intraperitoneal chemotherapy with oxaliplatin and 5-Fluorouracil. Pathologic examination showed only mucin and no atypical or neoplastic cells.

## 1 Introduction

Epithelial neoplasms of the appendix is the most common cause of PMP but it can also arise due to mucinous neoplasms of ovary, urachus, pancreas, colon, rectum or rarely stomach [1,2]. Complete Cytoreductive surgery (CCRS) and Hyperthermic Intra-peritoneal chemotherapy (HIPEC) has emerged the global standard of care for Pseudomyxoma Peritonei (PMP) over the past two decades. PMP is usually characterized by non-invasive nature of tumor and upfront CCRS is desired. However in certain patients the extensive nature of disease or medical conditions precludes surgery as treatment option.

Neoadjuvant chemotherapy in PMP has not yielded encouraging results with progression reported in 50 % of the study population [3,4]. Mesothelial cells of peritoneum create a significant plasma peritoneal barrier limiting the penetration of systemic drugs to the peritoneal tumor. Yonemura et al. reported the experience of neoadjuvant intraperitoneal therapy for gastric cancers with success [5,6]. Similarly we have been treating large volume or unresectable PMPs with Neoadjuvant Intra-peritoneal chemotherapy (NIPT). Here we present our experience of treating a 72 year old lady with PMP who had complete pathological response after NIPT and CCRS. This research work has been reported in line with the SCARE criteria [7] and informed consent was obtained from patient for publication of case report.

## 2 Case presentation

**Fig. 1:**
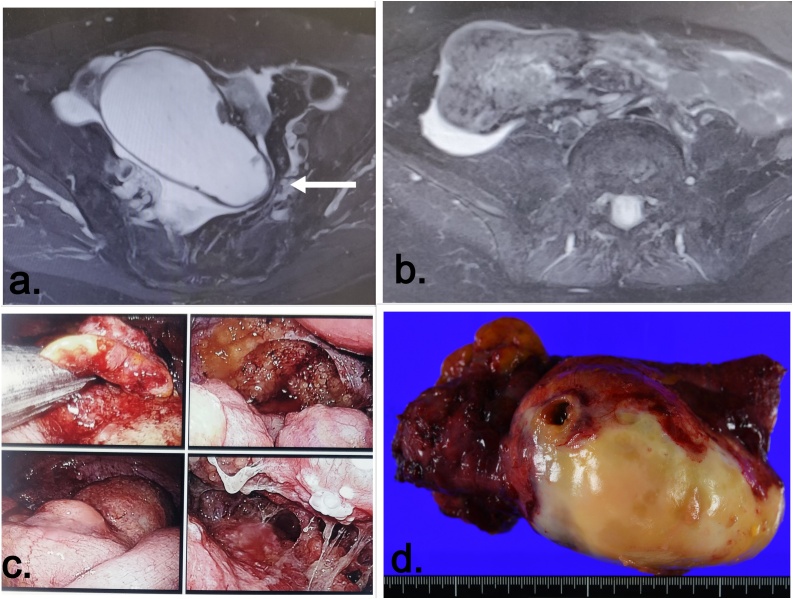
Initial (Pre- IP therapy) radiological, laparoscopic and pathological picture. a. MRI image with mucus filled appendix compressing the sigmoid lumen (arrow). b. Omental caking. c. Composite intra-operative picture showing omental mass and mucinous deposits all over abdomen. d. Gross specimen picture of perforated appendix.

A 72 year old lady presented with complaints of difficulty in passing stools. On evaluation by primary physician alarge appendicular mass compressing the sigmoid and the upper third of the rectum was found(Fig. 1.a). She was then referred to us and on initial evaluation in June 2018, she had BMI of 22.5 kg/m^2^ with Eastern Co-operative Oncology group Performance status (ECOG PS) Grade 1. Clinical examination revealed moderate ascites and mass in epigastrium probably due to omental caking. Serum tumor markers were as follows CA 125 – 73.8 IU/mL, CEA -7.1 ng/mL and CA 19-9- 453 U/mL. Imaging suggested a massively dilated appendix filled with mucinous material (15 × 7 × 7 cm) compressing the sigmoid colon. There was omental caking and multiple areas of loculated mucinous ascites suggesting Pseudomyxoma Peritonei (PMP) probably arising from appendicular pathology (Fig. 1.b).

Exploratory Laparoscopy was performed and following findings (Fig. 1.c) were noted – a. 2000 mL of ascites b. 15 × 20 cm Omental cake compressing on small bowel c. large perforated appendicular mass occupying the right iliac fossa and pelvis c. Mucinous tumor deposits in abdominopelvic quadrants 0,1,2,5,6,7 and 8 as described by Sugarbaker [8] and surgical Peritoneal carcinomatosis Index (PCI) was 19.

Ascites was evacuated and Ileo-caecal resection was performed through a limited right iliac fossa incision. Laparoscopic resection was technically not feasible due to the bulky disease and dense mucinous adhesions of cecum to posterior abdominal wall. Involvement of the cecum by tumor and tumor perforation precluded the performance of simple appendectomy (Fig. 1d). Following resection, Extensive Intraperitoneal lavage (EIPL) was done with 10 L of physiological saline and an Intra-peritoneal (IP) port system (Bard, Salt Lake City, USA) was placed in the abdomen with tip of catheter in *cul-de-sac*.

Gross pathological examination revealed perforation of tip of appendix (Fig. 1.d) and microscopic examination showed clusters of tumor cells in background of mucin with increased cellularity and high atypia. MIB-1 index was 22 % and a diagnosis of high grade mucinous appendicular neoplasm was made (Fig. 4.a).

**Fig. 2:**
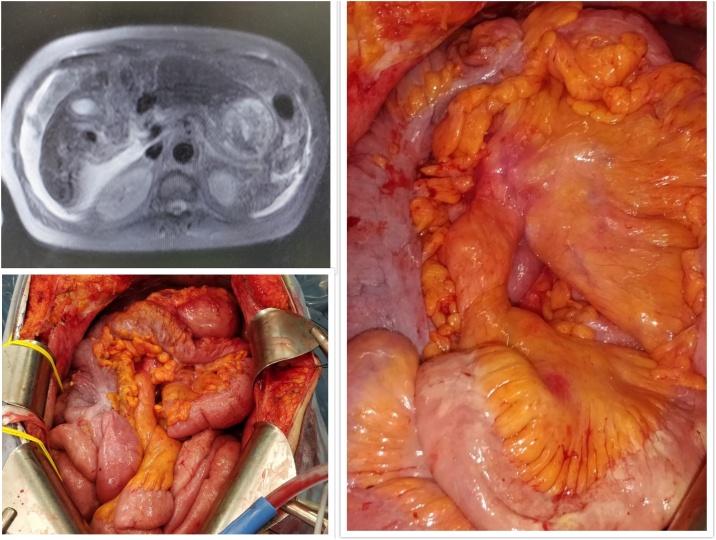
Post IP chemotherapy. a. MRI imaging showing resolution of omental mass. b. No tumor deposits in peritoneum and small bowel. c. Normal small bowel mesentery.

NIPT was initiated with Cisplatin 30 mg/ m^2^ and Docetaxel 30 mg/ m^2^ in 500 mL of physiological saline through IP port system every 3 weeks. After 6 cycles, an interim analysis showed clinical resolution of ascites, decrease in the serum levels of tumor markers and partial response in imaging. IP therapy was further continued with the same agents and overall she received 12 sessions of NIPT. There was complete clinical resolution of ascites and imaging resolution of omental mass (Fig. 2a). Tumor marker levels also normalized – CEA- 3.3 ng/mL, CA 125 – 25 IU/mL and CA 19-9 – 14.9 IU/mL.

In March 2020, she underwent a laparotomy and the following operative findings were noted (Fig. 2b& c) – a. Complete resolution of ascites. b. Omentum, stomach, small bowel and large bowel were normal with no evidence of tumor c. Liver, gall bladder, Spleen and uterus was normal with no evidence of tumor. d. Nodules in right paracolic gutter (2 × 2 cm) and pouch of douglas (multiple; each of size 1–1.5 cm). Both of the above contained only mucin and were negative for tumor metastasis in frozen section and e. rest of the peritoneal surfaces was clear of disease.

We performed CCRS with Total Anterior Parietal peritonectomy, Total omentectomy, lesser omentectomy, Pelvic peritonectomy including hysterectomy (sparing recto-sigmoid) and a completion right hemicolectomy. After EIPL with 10 L of physiological saline, HIPEC was performed with Oxalipatin (300 mg) and 5-Flurouracil (500 mg) in 4 L of saline by open “colosseum” technique. Intra-peritoneal temperature was maintained at 42.5–43.5 °C and HIPEC treatment time was 40 min.

**Fig. 3:**
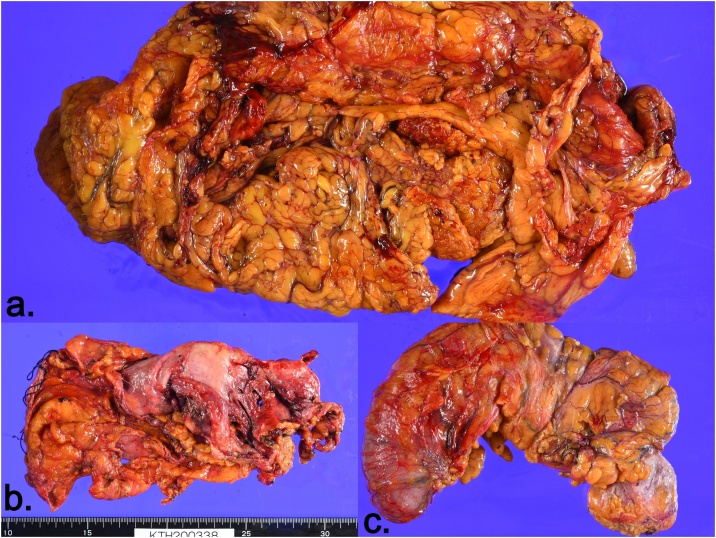
Gross pathological pictures. a. Omentum devoid of tumor and mucinous deposits, b. Pelvic Peritonectomy with en-bloc hysterectomy showing mucinous deposits, c. Completion right colectomy devoid of tumor and mucinous deposits.

**Fig. 4:**
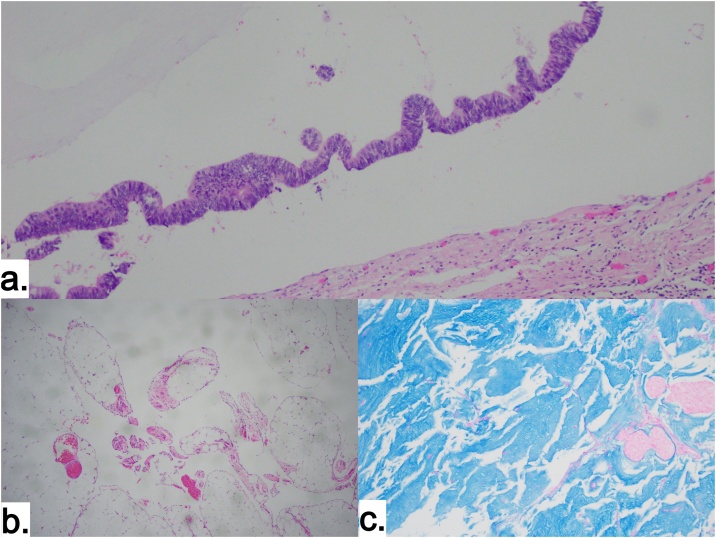
Histological picture. a. Appendix with high grade tumor cells after initial appendectomy. b. Microscopic examination (10x) of Omentum – without tumor cells (after CCRS) c. Acellular mucin in deposits (10x) with intense Alcian blue staining (after CCRS).

Pathologic examination revealed no viable tumor cells in omentum, peritoneum, uterus and colon. Only mucin deposition without cellularity was found on microscopic examination. Intense staining with Alcian blue indicating mucin deposition is shown in the picture (Fig. 3, Fig. 4). Post operatively she recovered well without complications and was discharged after 10 days in stable condition. She continues to be in regular follow-up with us.

## 3 Discussion

The CCRS with peritonectomy procedures and visceral resections as per tumor involvement was described by Paul Sugarbaker [9]. Since its inception in 1990s, multiple centers in the world have embraced CCRS and HIPEC for PMP and have reported long term survivals of 103–196 months with this treatment strategy [1,10]. In contrast to complete cytoreduction (CCR 0/1) the survival rates with incomplete cytoreduction (CCR-2/3) have been poor (5 year OS 24 %). Efforts to reduce tumor burden with systemic therapy have been tried in the past without much value.

IP chemotherapy as a neoadjuvant strategy has not gained popularity due to logistical inconvenience, lack of technical expertise and potential for local complications. However IP drug delivery has the advantage of achieving higher drug concentration in the peritoneal cavity. The advantage is usually expressed as the ratio of the area under the concentration-time curve (AUC) in the peritoneal fluid and AUC in the systemic compartment. This ratio is about 52–181 for docetaxel and 13–21 for cisplatin [11,12]. We have reported our experience of Neo-adjuvant Intra-peritoneal and systemic therapy (NIPS) and Neo-adjuvant Laparoscopic HIPEC (L-HIPEC) in gastric cancers with encouraging results [5,6]. We have also been following Neoadjuvant IP chemotherapy (NIPT) for high grade appendiceal neoplasms at our center.

In our approach, we perform appendectomy or ileo-cecal resections during initial laparoscopic assessment to remove perforated primary appendiceal tumor. This removes the source of mucin extrusion and further tumor deposition in the peritoneal cavity. Ascites however can re occur due to secretions from the established tumor deposits and ascitic drainage is performed prior to each session of NIPT to prevent the dilution of chemotherapeutic agent.

Our patient had complete resolution of tumor after 12 sessions of NIPT and the visceral resection was limited to right colonic and uterine resection. Upfront resection of this tumor would have potentially required additional bowel or visceral resections and would have incurred significant blood loss.

Local complications like infection, catheter blockade, abdominal pain, access issue after IP chemotherapy has been reported in 20–30 % of the subjects. Interestingly left colon or rectosigmoid resection increased the rate of port site infection in one of the reports [13,14]. Incidence of port related complications after NIPT in our center was around 12 % with the most common complication being catheter infection. Minor renal function derangements due to cisplatin can be found after 2–3 cycles and can be successfully managed with dose reduction. Catheter removal is required in 4% of our patients (unpublished data). The above facts suggests that with meticulous insertion and maintenance of IP port, sequential administration of IP chemotherapy is feasible resulting in strong suppression against peritoneal nodules over a long period of time.

In conclusion, Complete pathological response with IP chemotherapy is a rare occurrence in PMP especially with high tumor burden. While CCRS continues to be the major factor for prognosis, NIPT is a promising neoadjuvant strategy in patients who are poor candidates for upfront resection due to extent of disease or performance status, perhaps better than systemic therapy. It allows sufficient time for the patient to adjust to their clinical condition and receive better prehablitation before aggressive surgical therapy. Further clinical studies to ascertain its efficacy and to develop predictive markers for response assessment would be valuable.

## Declaration of Competing Interest

No Conflicts of Interest.

## Funding

No Funding received.

## Ethical approval

The procedure-specific consent, patient data and material of this study had reviewed and approved by the ethical review bodies of Kishiwada Tokushukai hospital, Osaka, Japan with ethic number 19–35 dated 11th November 2019.

## Consent

The consent for publication of case report was obtained from patient.

## Author contribution

Naveen Padmanabhan – Study conceptualization, Data collection, drafting manuscript.

Haruaki Ishibashi- Data collection and analysis.

Kazurou Nishihara- Data collection and analysis.

Shouzou Sako- Data collection and analysis.

Kanji Katayama- Data collection and analysis.

Satoshi Wakama- Data collection and analysis.

Yasuyuki Kamada- Data collection and analysis.

Yutaka Yonemura Study conceptualization, Data collection and review and finalization of manuscript.

All members have been reviewed the final version of manuscript.

## Registration of research studies

Not required as it is single case report and first in human study.

## Guarantor

Yutaka Yonemura.

Naveen Padmanabhan.

## Provenance and peer review

Not commissioned, externally peer-reviewed.
